# Comparison of determinants of research knowledge utilization by practitioners and administrators in the field of child and family social services

**DOI:** 10.1186/1748-5908-5-41

**Published:** 2010-06-03

**Authors:** François Chagnon, Louise Pouliot, Claire Malo, Marie-Joëlle Gervais, Marie-Ève Pigeon

**Affiliations:** 1 Department of Psychology, Université du Québec à Montréal, QC, Canada; 2 Chair CJM-IU-UQÀM on knowledge application, Université du Québec à Montréal, QC, Canada; 3 Centre jeunesse de Montréal Research Unit, Montréal, QC, Canada, School of Social Service, Université de Montréal, QC, Canada

## Abstract

**Background:**

An important gap exists between research production and its utilization. Few studies have examined the factors affecting knowledge utilization in the field of child and family social services.

**Methods:**

The objectives of the study are to describe knowledge utilization by child protection administrators and practitioners (N = 477) and to compare factors related to knowledge utilization by these two occupational groups. The study was conducted with an adapted version of the *Questionnaire sur l'utilisation des connaissances *(Knowledge Utilization Questionnaire). Factor analysis was used to collapse data collected on the questionnaire items. Factor score for each respondent served as independent variables in three separate multivariate regression analyses to explore variables likely to predict research-based knowledge utilization.

**Results:**

A minority of respondents (18%) report using on a frequent basis research-based knowledge in their practice. Relational capital between researchers and users and perceived usefulness of research based knowledge were the two factors most strongly related to utilization. There was a specificity in the factors associated with knowledge utilization according to occupational groups in child protection organizations. Use of active knowledge transfer strategies was associated with knowledge utilization by practitioners, while knowledge dissemination efforts played a more significant role for administrators.

**Conclusion:**

These results encourage both the use of strategies differentiated according to users and the intensification of interactions between users and researchers to foster research knowledge utilization.

## Background

Despite growing research productivity and accessibility to its products, studies consistently show a gap between the availability of scientific knowledge and its application [1-4]. Health researchers from the United States and the Netherlands have estimated that 30% to 45% of all patients are receiving inappropriate cares according to scientific evidences and from 20% to 25% of provided cares are unnecessary or potentially harmful [3].

Just as in the healthcare field, the desire to develop more solidly evidence-based practices in the child welfare field faces substantial obstacles [5-8]. In the province of Québec, youth centres form a network of organizations mandated to offer psychosocial services and protection to children in difficulty and their families. An estimated 100,000 young people receive youth centre services annually. The majority of such services are offered as part of protection measures and are aimed at children and families seriously affected by neglect, maltreatment, sexual abuse, abandonment, or severe behavior disorders.

Over the past years, the Québec Youth Centre network has invested significant financial and organizational resources in the implementation of evidence-based programs and practices. However, this transformation poses a challenge both in terms of bringing practitioners' clinical interventions more in line with evidence-based practices and encouraging the administrators of these organizations to make evidence-based strategic and administrative decisions [9].

Despite the large number of children and families receiving services from child and family social services in North America, very few studies have examined research knowledge utilization in such organizations. Indeed, most studies that have examined research utilization have been conducted in the health service sector with nurses [10,11]. From a recent systematic literature review carried out by our research team, we established the existence of N = 45 theoretical models of knowledge utilization, where among these 36 have been developed in the medical and nursing field. Only five of them dealt with the social intervention field, and none have been developed specifically in the child and family welfare context.

Given the different organizational culture of the medical field, which is highly hierarchical compared to child and family welfare sector of activity, one might wonder about the degree to which knowledge about determinants of research utilization in the health field can be applied to social and youth protection services. In addition, evidence is produced less frequently in social research due to the complexity of the variables under study and the more limited possibilities for controlled experimentation as compared to biomedical sciences. Finally, research utilization in clinical practice poses an even greater challenge in the child and family welfare field in that research-practice collaboration in child and family welfare is far more recent than in the healthcare field, and interventions are based for the most part on the clinical judgment and practical experience of practitioners and decision makers [12]. A number of studies have examined the attitudes of child and family welfare service providers toward adopting evidence-based practices. They indicated that different factors, namely organizational culture, work climate, organizational support, access to knowledge, and quality of training can influence the use of evidence-based practices in an intervention [13-16], as well as professional burnout and service provider turnover [5].

A recent survey by Children's Mental Health Ontario (CMHO) examines the perception of executive directors (N = 80) and practitioners (N = 483) of their organizations' ability to utilize research knowledge [17]. The survey revealed that fewer than 50% of respondents consider their organizations amenable to translate research knowledge successfully. There was general agreement among executive directors and practitioners concerning this issue. The results of a study conducted in the United Kingdom, with the participation of professionals from 50 child and family welfare services organizations, corroborate the organizations' role in supporting research and knowledge utilization by their staff. While a high proportion of the respondents (90%) considered that responsibility for the implementation of evidence-based practice should be shared by all, they stated that first there must be a strong leadership on the part of the administration [18].

To our knowledge, only one study has pertained to evaluate the real extent by which scientific knowledge is used by practitioners in child and family welfare services. The Australian study, conducted by Holzer *et al. *[8] with N = 495 professionals, showed that 62% of practitioners said they used research-based knowledge either often or always in their interventions. From a qualitative analysis applied on the content of 59 interviews conducted with respondents, the observations also suggested that two main factors influenced the use of research-based knowledge in clinical practice: organizational factors affecting support in access to and utilization of knowledge, and the concrete implications of knowledge for practice and its dissemination in formats adapted to users' needs [8]. However, no empirical study has examined or compared the determinants of research knowledge utilization for practitioners and administrators in child and family services.

The development of a better understanding of the conditions that contribute to the use of research-based evidence by practitioners and decision makers in the field of child and family welfare is vital to ensure better support for the translation of research-based knowledge into practice [9,19].

### Determinants of knowledge translation

Why is it so difficult to achieve high utilization of research-based evidence, and what are the key factors in this process? Studies show that organizational and individual determinants are involved in research knowledge utilization. On an organizational level, compatibility between types of knowledge available and the organization's need for new knowledge has been shown to foster the process of knowledge translation [20-22]. In this respect, the study of Barwick *et al. *[17] conducted in Ontario children's mental health services indicates that one of the major obstacles to knowledge translation is the lack of relevance of scientific information that is available to practitioners.

In addition to the relevance of available research, elements related to the organization itself may play an important role in knowledge utilization by members. Studies show that significant involvement by organizations throughout the research process, the implementation of a favorable organizational culture, and the presence of positive research values foster the acquisition and translation of research knowledge into practice [2,23-27].

Thus, an organization's receptivity to research knowledge utilization and its leadership in the domain have an important influence on user efforts to acquire, understand, and even participate in the development of knowledge; relatively unreceptive organizations are less likely to acquire research knowledge [18,23,24,28,29].

On an individual level, receptivity and attitudes of potential users towards research knowledge have been identified as being important factors in knowledge utilization [30,31]. Indeed, research results are often viewed by professionals as an incomplete source of knowledge. The integration of research knowledge into the knowledge developed by professionals and its integration into professional practice are particularly complex because the two areas of knowledge are the products of different cultures [32-34]. To be recognized as useful and applied effectively by professionals, such results must correspond to their clinical observations, practical knowledge, and relational skills [34].

Such difficulties may explain, in part at least, the significant gap that exists between the available research knowledge and its utilization in planning and intervention [1-3]. The use of research increases when the knowledge corresponds to users' needs and when users see the suitability of such knowledge to their own context [2,31,35-37].

In this respect, users' motivation to unfold efforts in acquiring and utilizing research knowledge may be influenced by their perception of potential risks in using research results in practice. Users' motivation to use research knowledge might be increased by the frequency and quality of contacts between researchers and users. 'Relational capital', or exchange mechanisms and level of trust existing between researchers and professionals, may be a determining factor in knowledge utilization because it contributes to bringing knowledge producers and users together and thus increases receptivity to the utilization of research knowledge [23,30,38]. Indeed, it has been shown that beneficial collaborative experiences with researchers improve users' attitudes towards research and increase the probability that they will engaged themselves in the knowledge utilization process [2,36]. Such collaborative experiences generally occur within the framework of exchange mechanisms of varying complexity, ranging from the simple exchange of written documentation to personal contact. Indeed, several studies have addressed the importance of such user-researcher exchange mechanisms in fostering knowledge utilization [2,28,39].

Although exchanges and relations between researchers and users of knowledge appear to play an important role in knowledge utilization, the notion of 'relational capital' is a concept that needs refinement in its definition to provide a better understanding of its relation to knowledge utilization and to clarify the conditions that foster the development of such capital between researchers and practitioners.

The theory of knowledge diffusion has played a central role in the development of theoretical models of knowledge translation, especially in the healthcare field [40]. Researcher efforts to disseminate research knowledge, especially when such efforts are intense and focused on mechanisms of interactive exchange with users, translate into products that are better adapted to users' needs and are better understood due to the greater amount of explanation that surrounded the dissemination, Studies highlight the fact that intensity of interactions between researchers and practitioners contributes to increased diffusion efforts by researchers and involvement by users [28]. However, diffusion efforts that are adapted specifically to targeted user groups by the producers of research knowledge are relatively uncommon [41].

### Targeting knowledge utilization

In addition to factors related to organizational characteristics and the receptivity of individuals able to influence knowledge utilization, choice of knowledge application strategies and users' targeted in the strategy itself seem to be crucial elements in knowledge utilization. Indeed, knowledge utilization needs and types may vary depending on the targeted users. Research indicates that knowledge utilization needs, as well as the appropriate messages and formats for transmitting knowledge, differ greatly depending on whether users are practitioners, program administrators, or political decision-makers [41-43]. For example, practitioners and administrators occupy different roles in child and family welfare. Practitioners intervene directly with the clientele, while administrators are responsible for making decisions related to service planning and administration. Thus, practitioners would be concerned with integrating research knowledge into their regular practice. This presupposes the organizational ability to support the transformation of practitioners' clinical practices over time in accordance with evidence-based practices [44,45]. Conversely, program administrators would be more concerned with finding specific information to help them make short-term decisions regarding the best action to take, *i.e.*, evidence-based decisions [9,41]. One of the key questions in promoting better utilization of research knowledge is to sort out whether knowledge-translation processes differ according to types of users, and if so what are the nature of processes at hand. Few studies have explored these questions. Two studies in the medical field have examined different forms of research knowledge utilization and compared knowledge-utilization processes engaged by different types of clinicians [46,47]. Results of these studies suggest differences in frequency and end-results of knowledge utilization depending on whether the knowledge is being used by nurse administrators, educators, or staff nurses.

In summary, research in the field of knowledge application suggests that research results utilization is determined by a complex set of variables comprised of organizational elements and other individual user-related elements. Exchange mechanisms and collaboration between researchers and professionals may play a determining role in this field. However, despite these advances, the specific roles of different elements and their interrelations remain unclear. While studies on the question to date provide a basic understanding of the factors at play in the equation, precise knowledge of the processes involved has yet to be developed.

This lack of knowledge is even more pronounced in child and family welfare, because the majority of studies on knowledge translation are conducted in the healthcare field. Moreover, while administrators and practitioners occupy different roles in child and family welfare services, no research conducted specifically in this field has examined whether the determinants of knowledge translation are different for the two groups of users.

## Methods

### Objective

The objective of this study was to examine determinants of research-based knowledge utilization by administrators of clinical services and professional practitioners in child and family social services organization. More specifically, we compared determinants of knowledge translation for two groups of professionals occupying distinct roles in the psychosocial services.

### Participants

The participants (N = 447) in this study were administrators of clinical services and practitioners working in the same youth centre establishment in Québec. Participants were recruited through a letter of invitation sent by the executive directors to the staff of their respective administrative sections. Potential participants were advised that they were free to accept or refuse the invitation. The project received ethical research and quality approval from the administration of the establishment.

### Administrators

All administrators of youth centre clinical programs (N = 102) were approached for the study. Of this number, 83 agreed to participate--34 women and 49 men--leading to a participation rate of 81%. Participants had on average 23.4 years of experience in their field (minimum = 5 years, maximum = 41 years).

### Practitioners

The practitioners solicited to participate in the study included all professional practitioners currently occupying full- or part-time positions providing child and family psychosocial services. Practitioners with part-time positions were included in the study because they represent 29% of the establishment's clinicians and are involved in the same activities as those with full-time positions. From the initial number of practitioners (N = 1,307), 442 agreed to participate. Questionnaires for which 15% or more of the answers were missing were eliminated, bringing the final number of respondents to 364 practitioners. Among these respondents, 243 were women and 120 were men, yielding 27.9% of the initial population. Participating practitioners had an average of 14.4 years of experience in their position (minimum = less than one year, maximum = 35 years).

### Final sample

The final sample comprised 83 administrators and 364 practitioners, representing 31.7% of the initial population. This rate is higher than that reported by a comparable child and family welfare study in Australia conducted by Holzer *et al. *in which the response rate was 8% [8]. The response rate for administrators in the current study (81%) is comparable to the rate in Barwick *et al. *[17], who reported a participation rate of 72.5% for administrators involved in child and family mental health services in Ontario and 12.2% for practitioners in the same services.

Analysis of participant distribution shows a greater proportion of women in the practitioner group and a greater proportion of men among the participating administrators (Chi square = 19.634, p < 0.01) which corresponds to the distribution generally found in the youth centre network. Number of years of experience is significantly higher in the administrator group (24.1 years) than in the practitioner group (14.4 years), (F 65.02, dl 1443, p < 0.001).

### Measures

An adapted version of the *Questionnaire sur l'utilisation des connaissances *(knowledge utilization questionnaire), developed by our research team, was used in this study [48]. The questionnaire was originally designed based on a study on knowledge utilization in the field of suicide prevention and proceeded from a critical analysis of previous measures used in the area of knowledge translation. The instruments consists of 77 items covering nine domains: relations with researchers; purposes and utilization of research knowledge; collaborations with researchers over the past two years; perceived efforts to foster knowledge translation; perceived efforts by researchers to adapt knowledge to users' needs; knowledge utilization over the past two years; effectiveness of communication mechanisms used between research and practice settings; perceived risks related to knowledge utilization; and organizational context. The instrument included a one-item scale as an index of the degree of research knowledge utilization by users. Respondents were asked to report, on a four-point scale ranging from 1 (never) to 4 (frequently) how frequently they used research results over the past two years. The rational behind the two-year reference period was to make sure that respondents' self-reported knowledge utilization was not unduly influenced by their recent experiences, occasional, or short-term collaboration in research projects. The rational was established upon discussion and consensus made with administrators in Québec youth centers. The temporal interval was similar to others studies pertaining to knowledge utilization in the healthcare field [47,49] and in public administration [38], ranging from one to five years, according to the studies reviewed.

For the purposes of the current study, 22 items from the original questionnaire were adapted to the context of child and family welfare. Factor analysis and varimax rotation were performed on items' responses due to modifications brought to the initial questionnaire [48]. A nine-factor solution was deemed adequate and explained 55.5% of the variance on instrument variables retained: usefulness of research knowledge; research knowledge dissemination efforts by researchers; organizational context; perceived cost; expectations of research knowledge; use of means of communication; attitudes towards collaboration with researchers; collaboration in research knowledge development; and efforts to acquire research knowledge. Internal reliabilities of the various scales used in the questionnaire were excellent, with Cronbach alphas ranging from 0.73 to 0.94.

### Procedure

Questionnaires were distributed to participants through their executive directors. A notice briefly explained the goals and procedures:

'The aim of this questionnaire is to examine how scientific knowledge is used in your organization, and to explore administrators' and practitioners' perceptions of the usefulness and quality of this knowledge. More specifically, the aim of this questionnaire is to learn about (1) your perceptions of the research sphere and scientific knowledge and (2) to get your opinion about elements that influence your utilization of scientific knowledge in your practice. All customary precautions will be taken to ensure that yours answers remain confidential. Only the members of the research team will have access to questionnaires and no information likely to identify you personally will be disseminated of published'

Pre-stamped pre-addressed envelopes accompanied the questionnaires and were return to the research team within three weeks of their distribution.

## Results

### Research knowledge utilization

Descriptive analyses of data distribution show that only 18% of administrators and practitioners said they had frequently used research knowledge in their work over the past two years (Table 1). A higher proportion of respondents from the practitioner group (38%) reported never or rarely having used such knowledge over the past two years, as compared to 29% of the respondents in the administrator group. We found no difference between respondents with full-time and part-time status with regard to frequency of research knowledge utilization.

**Table 1:** Research knowledge utilization by Québec youth centre respondents

	Administrators (N = 83)		Practitioners (N = 364)	
Over the past two years, I have used research knowledge in my work	Never	4%	Never	11%
	Rarely	25%	Rarely	27%
	A few times	53%	A few times	44%
	Frequently	18%	Frequently	18%

Chi Square = 6.24, dl.3,1, p = 0.10

### Determinants of Knowledge Utilization

A series of three multiple regression analyses, one standard and two hierarchical types, was carried out; The analyses aimed to determine the contribution of a set independent variables (IVs), some obtained through factor analysis (*i.e.*, group, usefulness of research knowledge, efforts to collaborate in the development of research knowledge, research knowledge dissemination efforts by researchers, organizational context, perceived cost of research knowledge translation into practice, expectations of research knowledge, use of means of communication, attitudes towards relations with research, and efforts to acquire research knowledge) to the prediction of knowledge utilization. In the analyses, factor scores for each respondent served as IVs. Variable inflation factors (VIFs) were computed for each predictor variable to detect multi-co-linearity. As a guideline, a VIF > 10 indicated a problematic co-linearity [50]. Statistical tests indicated that multi-co-linearity was not a significant problem The maximum VIF among our predictor variables was approximately 1.

An initial analysis was conducted with all respondents included. Eight out of ten independent variables contributed significantly to the prediction of research knowledge utilization in practice: collaboration in research knowledge development (sr^2 ^= 0.29); perceived usefulness of research knowledge (sr^2 ^= 0.25); perceived efforts by researchers to disseminate research knowledge (sr^2 ^= 0.18); personal efforts to acquire research knowledge (sr^2 ^= 0.17); favorable attitudes towards relations with researchers (sr^2 ^= 0.13); use of means of communication (sr^2 ^= 0.14); organizational context (sr^2 ^= 0.09); and perceived cost of knowledge utilization (sr^2 ^= -0.10). Together, these eight variables accounted for 29% of the variation in the prediction of research knowledge utilization in practice, (R^2 ^= 0.29, F (10, 436) = 17.54, p < 0.001). Table 2 provides a summary of regression coefficients.

**Table 2:** Standard multiple regression for respondents as a whole

Variables	Utilization (DV)	B	Β (standardized)	**sr**^**2 **^**(unique)**
Group	0.08	-0.13	-0.06	-0.06

Attitudes towards collaboration with researchers	0.16***	0.13	0.13**	0.13

Collaboration in research development	0.30***	0.28	0.30***	0.29

Organizational context	0.10*	0.08	0.09*	0.09

Efforts to acquire knowledge	0.19***	0.17	0.17***	0.17

Perceived costs	-0.09*	-0.08	-0.10*	-0.10

Expectations of research	0.05	0.04	0.04	0.04

Dissemination efforts by researchers	0.21***	0.16	0.18***	0.18

Use of means of communication	0.16***	0.13	0.14***	0.14

Usefulness of knowledge	0.27***	0.22	0.26***	0.25

	Intercept =	0.775		

				R^2 ^= 0.29

				adjusted R^2 ^= 0.27

				R = 0.54***

* p ≤ 0.05

**p ≤ 0.01

***p < 0.001

While according to this analysis, the 'group' variable does not appear to be a significant predictor of research knowledge utilization, bivariate correlational analyses nonetheless showed a correlation with such utilization. To verify whether different variables could predict knowledge utilization by administrators or practitioners, separate analyses were conducted for each occupational group.

### Multiple standard regression analysis on practitioner answers

Analysis showed that seven of the nine independent variables contributed to the prediction of research knowledge utilization by practitioners (Table 3): collaboration in research knowledge development (sr^2 ^= 0.27); perceived usefulness of research knowledge (sr^2 ^= 0.23); efforts made to acquire research knowledge (sr^2 ^= 0.18); use of means of communication (sr^2 ^= 0.16); perceived efforts by researchers to disseminate research knowledge (sr^2 ^= 0.15); attitudes towards relations with researchers (sr^2 ^= 0.14); and organizational context (sr^2 ^= 0.10). Together, these seven variables accounted for 28% of variability (26% adjusted) in the prediction of research knowledge utilization in practice, (R^2 ^= 0.28, F (9, 354) = 15.26, p < 0.001; see Table 4).

**Table 3:** Standard multiple regression for the practitioner group, based on variables measured in the youth centre study on knowledge translation

Variables	Utilization (DV)	B	β	**sr**^**2 **^**(unique)**
Attitudes towards collaboration with researchers	0.17***	0.14	0.14**	0.14

Collaboration in research development	0.28***	0.27	0.27*** -0.44***	0.27

Organizational context	0.10*	0.09	0.10*	0.10

Efforts to acquire knowledge	0.21***	0.18	0.18***	0.18

Perceived costs	-0.08	-0.07	-0.08	-0.08

Expectations of research	0.06	0.05	0.06	0.06

Dissemination efforts by researchers	0.20***	0.14	0.15*** -0.36***	0.15

Use of means of communication	0.18***	0.14	0.16***	0.16

Usefulness of knowledge	0.26***	0.21	0.24*** -0.43***	0.23

	Intercept =	0.466		

				R^2 ^= 0.28

				adjusted R^2 ^= 0.26

				R = 0.53***

*p ≤ 0.05

**p ≤ 0.01

***p ≤ 0.001

**Table 4:** Standard multiple regression for the administrator group, based on variables measured in the youth centre study on knowledge translation

Variables	Utilization (DV)	B	β	**sr**^**2 **^**(unique)**
Attitudes towards collaboration with researchers	0.05	0.03	0.03	0.03

Collaboration in research development	0.34***	0.38	0.44***	0.41

Organizational context	0.01	0.05	0.05	0.04

Efforts to acquire knowledge	0.05	0.08	0.09	0.09

Perceived costs	-0.06	-0.14	-0.16	-0.15

Expectations of research	-0.02	-0.06	-0.06	-0.06

Dissemination efforts by researchers	0.28**	0.30	0.36***	0.34

Use of means of communication	-0.02	-0.03	-0.03	-0.03

Usefulness of knowledge	0.35***	0.35	0.43***	0.41

	Intercept =	0.719		

				R^2 ^= 0.42

				adjusted R^2 ^= 0.35

				R = 0.65 ***

*p ≤ 0.05

**p ≤ 0.01

***p ≤ 0.001

### Multiple standard regression analysis on administrator answers

The model that emerged for administrators was less complex. Indeed, only three of the independent variables considered in the study contributed significantly to the prediction of knowledge translation by administrators: collaboration in research knowledge development (sr^2 ^= 0.41); perceived usefulness of research knowledge (sr^2 ^= 0.41); and research knowledge dissemination efforts on the part of researchers (sr^2 ^= 0.34). Together, these three variables accounted for 42% of variability (35% adjusted) in the prediction of research knowledge utilization, (R^2 ^= 0.42, F (9, 73) = 5.83, p < 0.001; Table 4).

### Discussion

This study shows that research utilization in child and family welfare service organizations is uncommon. Indeed, only 18% of administrators and practitioners said they frequently used research knowledge at work. Utilization was particularly low among practitioners. These rates are similar to those observed by Barwick *et al. *[17] in child mental health services in the Canadian province of Ontario, and they are lower than those reported in Holzer *et al. *[8] of child and family welfare services. These results underlined the need to develop and implement strategies that foster knowledge translation in child and family welfare services organizations.

Recent research suggest that relational capital, or the relationships and bonds of trust that develop through collaboration between research and practice, may be a key concept in the process leading to research knowledge utilization [30,38]. Our results point in this direction, because collaboration and involvement with researchers in the development of research knowledge is the most important factor in predicting knowledge utilization by respondents as a whole. A finer description of these results affords a better understanding of the concept of relational capital. Relational capital was operationalized in this study by two factors: a behavioral factor measuring user's involvement in collaborative experiences with researchers to develop knowledge, and another more subjective factor measuring attitudes towards collaboration with researchers. While real involvement in collaborative efforts is a better predictor of knowledge utilization, favorable attitudes towards collaboration with researchers are also positively associated with research knowledge utilization.

In this study, the perceived usefulness of research-based knowledge proved to be the second most important factor in predicting knowledge utilization (r^2 ^= 0.25). This agrees with the results of previous studies that found research knowledge more likely to be used when it corresponds to users' needs and when users see its applied value to their practice [2,31,34,35,37].

Collaborative experiences with researchers and involvement in the development of research knowledge may be intervening variables that bolster perceived usefulness of knowledge in practice and its desired end-result knowledge utilization. These two factors have been found to be closely associated in past research [2,23,28,30,35,36,51,52]. Furthermore, it may be argued that frequent exchanges and linkage among practitioners, administrators, and researchers promote trust among the groups and sustain collaboration among these partners, with both yielding to the development of targeted research questions and approaches more in line with practitioners' needs. This, in turn, can have a positive impact on the perceived value of research knowledge to practice and, as a result, increase its use. These results highlight the importance of supporting the process of collaboration between researchers and practical settings to foster research knowledge utilization.

It is interesting to observe that, contrary to the perceived usefulness of results, expectations of research do not contribute to predicting knowledge utilization. One explanation could be that it is a factual understanding of the practical implications of research knowledge that encourages utilization rather than initial expectations. This hypothesis is supported by the fact that 'perceived usefulness of research results' was a significant predictor of research utilization in our sample. Another explanation could be that, given the low research utilization by the administrators and practitioners in our study, their expectations about research may have been relatively ill-defined to begin with.

**Figure 1:**
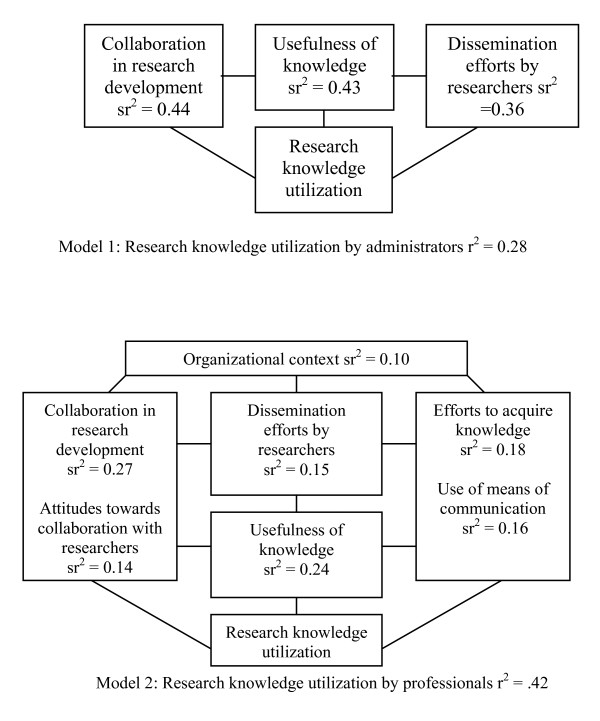
**Modelization of factors related to knowledge utilization, by respondent group**. Model 1: research knowledge utilization by administrators, (R^2 ^= 0.42, F (9, 73) = 5.83, p < 0.001. Model 2: research knowledge utilization by professionals (R^2 ^= 0.28, F (9, 354) = 15.26, p < 0.001).

Analysis of the data by respondent group affords a better understanding of associated relations among variables, and shows that the factors tied to the prediction of knowledge utilization vary by group. While in both groups real collaboration with research is the most important factor for predicting knowledge utilization, specific factors seems more important from one group to the other (Figure 1).

In the service administrator model, only three variables predict knowledge utilization. While participation in collaborative experiences with researchers is the most important predictor, perceived usefulness of knowledge from the administrator's perspective and the efforts attributed to researchers in developing and disseminating knowledge adapted to users' needs contribute almost equally in the explanation of knowledge utilization by administrators. These results also underline the importance of identifying formats suited to users' needs and suggest that researcher efforts at knowledge dissemination, or the recognition of such efforts in practical settings, is particularly crucial in fostering knowledge utilization by administrators of social service programs.

In the practitioner model, collaboration with researchers and perceived usefulness of research knowledge again constituted the two most important predictors of knowledge utilization. However, the practitioner approach to knowledge utilization is characterized by two active knowledge-seeking strategies. Personal efforts to acquire research knowledge comprise the third most important predictive factor for the practitioner group, although this factor is not a significant contributor in the administrator model. Further, the medium of communication used to obtain research knowledge is shown to be a significant factor in the explanatory practitioner model. Here again, perceived efforts on the part of researchers to disseminate knowledge adapted to users' needs contribute in the prediction of knowledge utilization, although to a far lesser degree than in the administrator model. Finally, the existence of favorable conditions in the organizational context also contributes significantly and specifically to the explanatory model of knowledge utilization by practitioners. Once again, these observations strengthen our previous hypothesis, and are in accordance with those of prior studies. Together, these results support the importance of adopting specific strategies according to user group to foster knowledge utilization [41-43].

### Limitations

Knowledge translation is a relatively new field of study in many ways. The construct draws on numerous concepts, including motivation, attitude, expectation, perception, and dissemination; the contours of this precise field of inquiry are somewhat ill-defined for the moment in the literature. In addition, knowledge translation, like any social behavior in general, is not secluded from social, cultural, and individual factors (such as personality traits) surfacing the contours of the problem at hand. In addition, while measurements were performed on some characteristics of the work organization context, it remains that organizational culture was not part of the variables examined in this exploratory study. This variable should be considered in future research. Thus, the results of this exploratory study must be considered as an initial step towards a better empirical understanding of knowledge translation processes among decision-makers and practitioners in the field of child and family welfare services. However, it is obvious that other factors also should be examined in this complex equation. The survey approach used in this study made possible a further step in the clarification of the relative contributions of factors related to the organizational context, users, and researchers. However, measurement of research knowledge utilization continues to be general and exploratory. A more in-depth study based on different specific knowledge utilization situations could provide a better understanding of the role these factors play in the translation of research knowledge. In addition, the observations in this study were collected from practitioners and administrators working in the same youth centre establishment and could prove different in another establishment. Nevertheless, the size of the sample that participated in this study guarantees stability in the results in the event of future replications.

## Summary

This exploratory study suggests that research knowledge utilization in child and family welfare services is rare. Relational capital between professionals and researchers is based on both effective collaboration and favorable attitudes towards research, and was found in this study to be the variable most strongly associated with research knowledge utilization. The results also put forward the significance of clarifying and reinforcing the perceived usefulness of research results to practice for administrators and practitioners alike. Linkage and sustained interaction between research and practice could foster the production of knowledge better targeted to users, improve the perceived value of results, and encourage their utilization by administrators and practitioners. In addition to these variables, distinct factors also explain knowledge utilization, notably dissemination efforts by researchers as reported by administrators, and the use of active strategies by practitioners. These results are even more important as they provide empirical support for the recommendation advocating collaboration among practitioners, administrators, and researchers in elaborating research priority questions and developing our understanding of the practical implications of research knowledge. While this exploratory study supports the relevance of developing specific strategies based on the needs of practitioners and administrators to improve research knowledge utilization, further research on this question is essential. Measuring knowledge utilization in specific situations and using comparison of administrators and practitioners would provide a better understanding of the elements associated with better knowledge utilization for these two groups of child and family welfare professionals.

## Competing interests

The authors declare that they have no competing interests.

## Authors' contributions

FC and CM designed the study. LP and FC conducted the analysis and participated in the drafting of the manuscript. MJG contributed conceptually to the literature review and commented earlier drafts of the manuscript. MEP contributed to the data collection. All the authors made comments and they approved the final manuscript.

## Authors' information

FC is professor in the Department of Psychology at the Université du Québec à Montréal (UQÀM), holder of the CJM-IU-UQÀM Study Chair on knowledge translation in the field of child and family welfare, and researcher at the Centre jeunesse de Montréal-Institut Universitaire. LP is associate researcher with the CJM-IU-UQÀM Chair. CM is researcher at the Centre jeunesse de Montréal-Institut Universitaire and associate professor with the School of Social Service at the Université de Montréal. M-JG is doctoral student at UQÀM and research officer for the CJM-IU-UQÀM Chair. M-ÈP is a doctoral student at UQÀM and associate with CJM-IU-UQÀM Chair.
